# Validation and Comparison of Two Methods to Assess Human Energy Expenditure during Free-Living Activities

**DOI:** 10.1371/journal.pone.0090606

**Published:** 2014-02-28

**Authors:** Panagiota Anastasopoulou, Mirnes Tubic, Steffen Schmidt, Rainer Neumann, Alexander Woll, Sascha Härtel

**Affiliations:** 1 House of Competence - hiper.campus, Karlsruhe Institute of Technology, Karlsruhe, Germany; 2 Department of Sport and Sports Science, Karlsruhe Institute of Technology, Karlsruhe, Germany; CNRS, France

## Abstract

**Background:**

The measurement of activity energy expenditure (AEE) via accelerometry is the most commonly used objective method for assessing human daily physical activity and has gained increasing importance in the medical, sports and psychological science research in recent years.

**Objective:**

The purpose of this study was to determine which of the following procedures is more accurate to determine the energy cost during the most common everyday life activities; a single regression or an activity based approach. For this we used a device that utilizes single regression models (GT3X, ActiGraph Manufacturing Technology Inc., FL., USA) and a device using activity-dependent calculation models (move II, movisens GmbH, Karlsruhe, Germany).

**Material and Methods:**

Nineteen adults (11 male, 8 female; 30.4±9.0 years) wore the activity monitors attached to the waist and a portable indirect calorimeter (IC) as reference measure for AEE while performing several typical daily activities. The accuracy of the two devices for estimating AEE was assessed as the mean differences between their output and the reference and evaluated using Bland-Altman analysis.

**Results:**

The GT3X overestimated the AEE of walking (GT3X minus reference, 1.26 kcal/min), walking fast (1.72 kcal/min), walking up−/downhill (1.45 kcal/min) and walking upstairs (1.92 kcal/min) and underestimated the AEE of jogging (−1.30 kcal/min) and walking upstairs (−2.46 kcal/min). The errors for move II were smaller than those for GT3X for all activities. The move II overestimated AEE of walking (move II minus reference, 0.21 kcal/min), walking up−/downhill (0.06 kcal/min) and stair walking (upstairs: 0.13 kcal/min; downstairs: 0.29 kcal/min) and underestimated AEE of walking fast (−0.11 kcal/min) and jogging (−0.93 kcal/min).

**Conclusions:**

Our data suggest that the activity monitor using activity-dependent calculation models is more appropriate for predicting AEE in daily life than the activity monitor using a single regression model.

## Introduction

Physical activity is an important determinant of health, and a lack of physical activity increases the risk of developing diseases and conditions including coronary heart diseases, Type 2 diabetes and colon and breast cancer and decreases life expectancy [1]. In recent years, a large research effort has been put into developing effective physical activity measures for disease prevention and therapy. Accurate assessment of physical activity and its outcomes is a prerequisite for evaluating the efficacy of novel prevention and rehabilitation interventions [2], [3] and for monitoring physical activity profiles of patients with limited mobility such as patients with multiple sclerosis, Parkinson’s disease or patients undergoing rehabilitation [4], [5].

Daily physical activity is typically assessed using energy expenditure (EE) estimates where activity-related energy expenditure (AEE) is the most varying component of total energy expenditure (TEE). The amount of the daily AEE may vary from 15% TEE in less active persons to 65% TEE in very active persons [6] and can therefore be used as dimension for the assessment of physical activity. The gold standards for measuring EE are indirect calorimetry in laboratory settings [7] and the doubly labeled water method for field testing [8]. Alternative methods for physical activity assessments include questionnaires or diaries, and accelerometers. The doubly labeled water method is very costly and cumbersome and does not resolve data by time, and questionnaires and dairies are subjective measures with moderate accuracy of measuring physical activity [9]. In contrast, accelerometry is suitable for field testing and has the potential for accurately measuring daily physical activity, and hence has become the most frequently used technique for assessing daily physical activity [10]. Accelerometer based devices use the recorded acceleration for identifying postures, classifying between types of daily activities, measuring number of steps, identifying gait patterns and identifying normal or abnormal movements (e.g. falls) [11]. Moreover, most commercially available accelerometers also estimate the EE for daily activities.

Accelerometers can be designed as uniaxial, multi-axial or as multi-sensor systems. Previous studies have investigated the validity of different commercial acceleration-based devices for assessing EE in healthy [12], [13] and unhealthy subjects [14] and whether multi-axial accelerometers are superior in quantifying physical activity compared to uniaxial [15] or multi-sensor systems [16]. The main disadvantage of many current acceleration-based activity monitors is that they are based on the assumption of a linear association between the acceleration (usually in form of activity counts) and EE across all types of activities and thus use single regression equations for predicting EE. However, predicting EE from a single regression equation often results in overestimating the EE during low intensity activities and underestimating EE during all other activities [17]. To overcome this problem, separate regression models can be used for each type of activity. In fact, using this activity-based approach EE estimation is more accurate than using a single regression based method [18], [19].

The purpose of this study was to determine the accuracy of measuring AEE during the most common daily activities for the two acceleration-based activity monitors GT3X (single regression model) and move II (activity-based regression models) compared to indirect calorimetry (IC).

## Materials and Methods

### Subjects & Ethics Statement

Nineteen healthy subjects (11 men and 8 women) participated in this study after providing written informed consent. Subjects with a large range in age, body characteristics and physical conditions and both sexes were included in this study to represent a sample that roughly corresponds to the general adult population. Only subjects who were between 18 and 55 years old were included. Exclusion criteria were chronic diseases, body impairments and medication intake.

This study was exempt from full ethics review by the Ethics Committee at the Karlsruhe Institute of Technology. All subjects gave written informed consent prior to participation. The study was conducted in accordance with the Declaration of Helsinki.

### Anthropometric Measurements

Prior to testing, subjects’ height and mass were measured (without shoes and in light clothing), using a stadiometer and a calibrated physician’s scale (Seca GmbH, Hamburg, Germany), respectively. Body mass index (BMI) was calculated as the subject’s mass (in kg) divided by the height squared (in m^2^). Descriptive data of all subjects are listed in Table 1.

**Table 1:** Descriptive characteristics of the participants (mean, SD, minimum, maximum).

	Women (N = 8)				Men (N = 11)			
	Mean	SD	Min	Max	Mean	SD	Min	Max
Age [years]	30.8	8.6	23.0	46.0	30.6	9.0	22.0	51.0
Height [cm]	167.3	4.2	161.0	173.5	178.7	7.3	166.6	192.5
Mass [kg]	65.2	9.0	51.8	78.8	80.3	12.3	64.0	103.2
BMI [kg/m^2^]	23.3	3.0	19.6	28.7	25.1	3.4	21.2	33.4
RMR [kcal/d]	1631.6	197.1	1330.0	1840.0	2163.5	291.3	1904.0	2773.0

BMI – body mass index; RMR – resting metabolic rate.

### Procedures

First, the resting metabolic rate (RMR) was measured. Subjects were equipped with the indirect calorimeter. During RMR-measurements, the subjects were at complete rest in supine position and were asked to relax, to refrain from any movements and from talking, and to lightly breathe. RMR was extracted from a 5-minute steady state period without high fluctuations in the VO_2_, VCO_2_, and respiratory quotient (RQ). This was automatically performed from the IC software. The RMR-measurement ended automatically after completing a 5-minute steady state period and lasted between 30 and 45 minutes.

Subsequently, the subjects performed a series of different predefined daily indoor and outdoor activities (Table 2) including walking at different speeds, walking up- and down-stairs and crossing a sloped pedestrian bridge (representing walking up- and downhill). To assess the validity of the two devices for measuring EE in free-living activities, we aimed at creating situations similar to those found under habitual conditions and asked each subject to perform the activities at his/her normal intensity. To avoid influencing the intensity of the activity, the investigator only gave instructions when to start and stop.

**Table 2:** Study procedure.

Activity	Duration	Distance	Place
Sitting	5 min	–	indoor
Standing	5 min	–	indoor
Slow walking		415 m	outdoor
Fast walking		415 m	outdoor
Jogging		2×415 m	outdoor
Walkingup−/downhill		4×130 m	outdoor
Walking stairsup/down		3 floors	indoor

Between consecutive types of activities, subjects were asked to rest. This was necessary to accurately assess AEE for each activity and to ensure that the subjects did not experience any fatigue. Because different recovery times may be required following different activities and different intensities, the break was defined as the time needed by the subject to reach 20% above his/her resting heart rate. Overall, data collection took around 75 minutes including all transitions between the activities and breaks.

### Devices

All subjects simultaneously wore the portable IC MetaMax 3B (Cortex Biophysik, Leipzig, Germany) as criterion measure of the EE, the GT3X (ActiGraph Manufacturing Technology Inc., Pensacola, FL, USA) and the move II (movisens GmbH, Karlsruhe, Germany) activity sensors attached to their waist above the right anterior axillary line according to the manufacturers’ recommendations. The heart rate was monitored using a Polar Activity Watch (Polar Electro Oy, Kempele, Finland). Prior to each trial, all devices were initialized and synchronized using their respective software.

#### Indirect calorimetry

The MetaMax 3B was calibrated before each test according to the manufacturer’s guidelines. The IC consists of a face mask, a measurement module and a battery module. The two modules have the same size (12×11×4.5 cm^3^) and are attached to the chest by a harness. The entire system weighs approximately 570 g and can operate remotely for up to 15 hours. The measured data is transmitted wirelessly to a laptop and can be further analyzed using the software MetaSoft (Cortex Biophysik, Leipzig, Germany). The validity and reliability of the MetaMax 3B compared with the Douglas bag method and another validated gas analysis system (Jaeger Oxycon Pro system) have been previously reported [20].

#### ActiGraph – GT3X

The GT3X activity sensor consists of a three-axial acceleration sensor (adxl335, Analog Devices, Boston, USA; range: ±3 g; sampling rate: 30 Hz; resolution: 12 bit). The sensor weighs 27 g, measures 3.8×3.7×1.8 cm^3^, can be worn either on the hip or on the wrist and allows measurements for up to 21 days. The recorded data is saved as activity counts on a 4 MB flash memory and transferred to the computer via standard USB 2.0 interface.

#### movisens GmbH - move II

The move II activity sensor consists of a three-axial acceleration sensor (adxl345, Analog Devices; range: ±8 g; sampling rate: 64 Hz; resolution: 12 bit) and an air pressure sensor (BMP085, Bosch GmbH; resolution: 0.03 hPa; sampling rate: 8 Hz). The sensor weighs 32 g, measures 5.0×3.6×1.7 cm^3^, can be attached at different locations (hip, wrist or chest) and allows measurements for up to 7 days. The recorded raw data is saved on a 2 GB micro SD card and transferred to the computer via standard USB 2.0 interface.

### Data Processing

The data from the IC were analyzed using the associated software (MetaSoft). The IC was used both to collect the reference data for the EE and to set the start- and stop-markers for the different activities. For each activity, steady state EE was identified, and the corresponding EE values were averaged and used for subsequent analyses. This procedure was repeated for all subjects and the following activities: walking, fast walking and jogging. Because of the short durations of all other activities (walking up−/downhill, ascending and descending stairs), steady state was not reached during these activities, and the mean EE was calculated for each activity and used as reference. The AEE for each activity was calculated by subtracting the RMR from the EE.

The raw data from the GT3X in form of counts were analyzed using the ActiLife 5 (ActiGraph Manufacturing Technology Inc., FL., USA) software. The device was set to record every second (epoch). The subject’s mass was entered prior to data collection, and the software was set to calculate AEE per second using a “vector magnitude” algorithm. A more detailed description on estimating AEE using the GT3X can be found elsewhere [21].

The raw data from the move II were analyzed using the associated software DataAnalyzer (movisens GmbH, Karlsruhe, Germany). The output sampling rate was set to 1 sec. The subject’s physical characteristics (age, height, mass and sex) were entered prior to data collection, and the software estimated AEE. A more detailed description on estimating EE using the move II has been published previously [22].

All data were imported into MS Excel (Microsoft Corporation, Redmond, USA) and synchronized for further statistical analyses. The second by second AEE estimates for both devices were converted into kcal/min, and the data were averaged to define the AEE rate for each subject and each activity expressed as kcal/min.

### Statistical Analysis

All statistical analyses were performed using the open source computing language and statistics package R 2.13.2 and SPSS version 17.0 (SPSS Inc., Chicago, IL, USA). The accuracy of the two devices for predicting AEE was defined as the mean difference (in kcal/min and in percent) between estimated AEE and the reference AEE from IC for each device and each activity. To assess the agreement of the two measuring devices with respect to the reference, a Bland–Altman analysis [23] was performed. The Bland–Altman plots for each device and each activity were calculated and the measurement errors of both devices were plotted against their bias. A zero bias represented no difference between estimated and reference AEE, a negative bias (estimated AEE minus reference AEE) indicated an underestimation of AEE by the monitoring device, and a positive bias corresponded to an overestimation of AEE by the monitoring device. The limits of agreement, which are defined as the mean difference (bias) ±1.96 times the standard deviation of the errors, are also shown in the plots. The smaller the range between these two limits the more accurate is the device.

## Results

**Figure 1:**
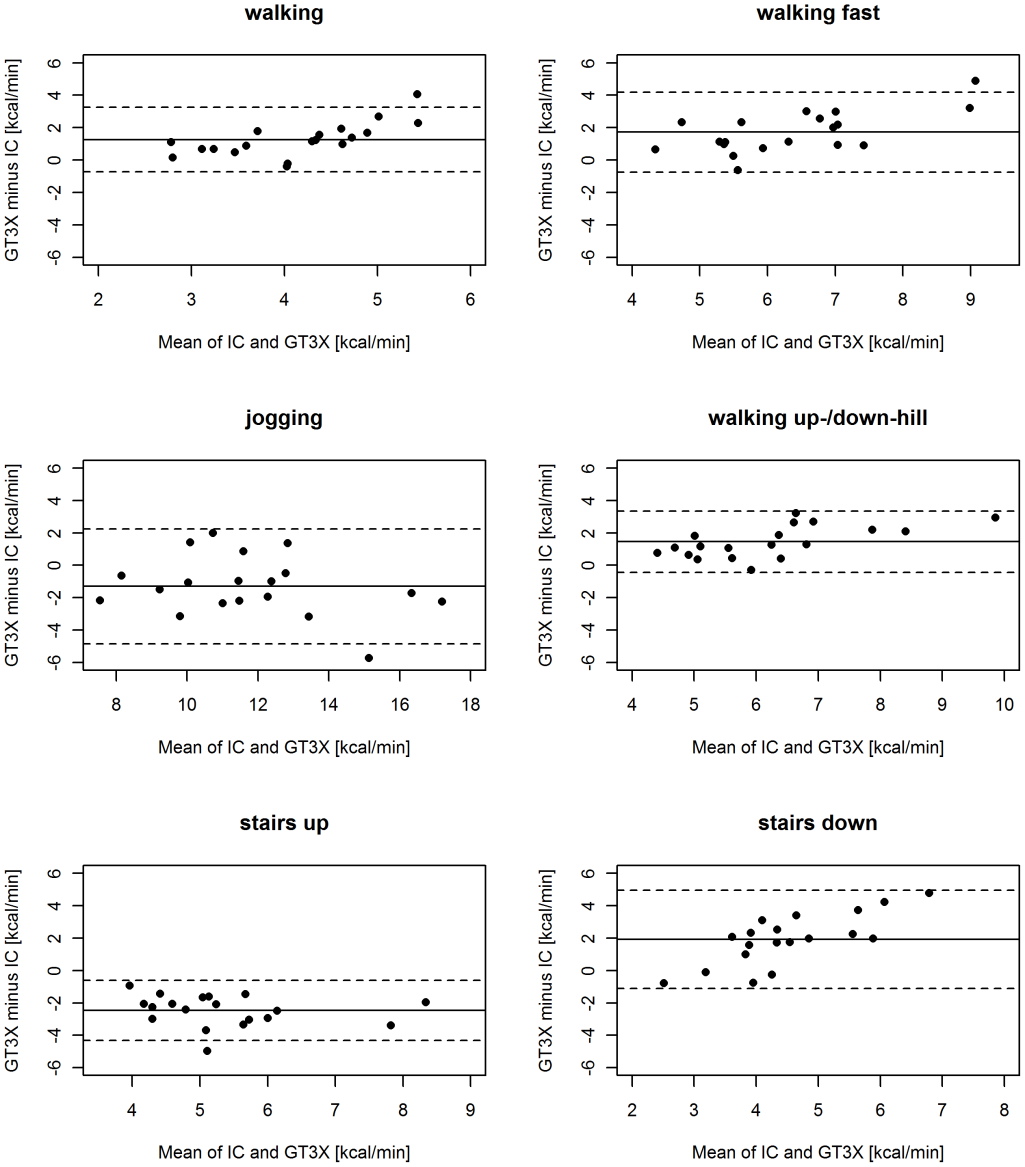
Bland–Altman plots for GT3X. The solid lines represent the mean bias between estimated and reference AEE, and the dashed lines represent the limits of agreement (±1.96 SDs).

**Figure 2:**
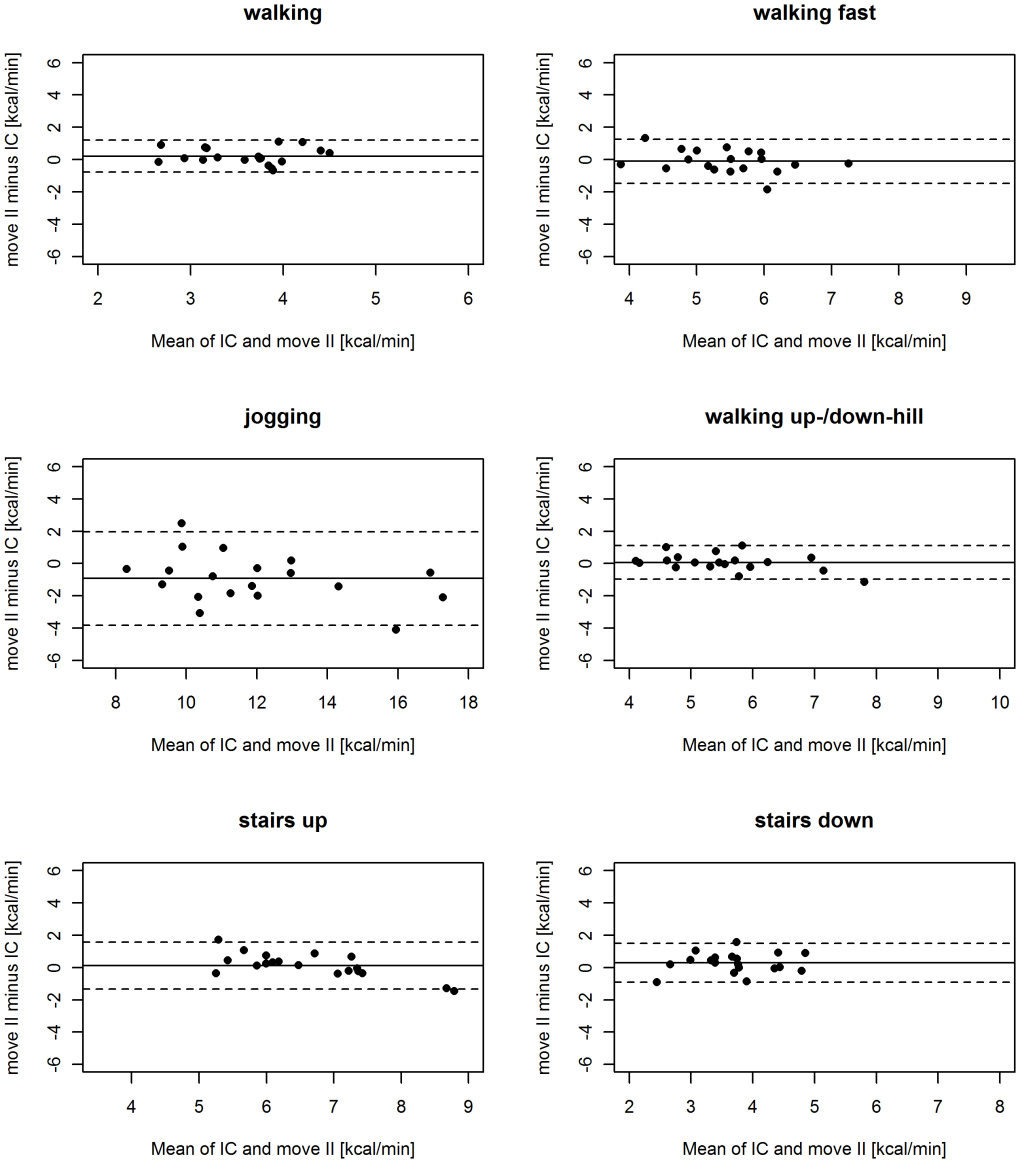
Bland–Altman plots for move II. The solid lines represent the mean bias between estimated and reference AEE, and the dashed lines represent the limits of agreement (±1.96 SDs).

The mean and percent differences between AEE measured using the activity monitors and that measured using IC (reference) differed between activities (Table 3). The Bland-Altman plots for the GT3X and the move II are shown in Figures 1 and 2, respectively. Overall, the differences relative to the reference values were 1.5 to 15 times larger for the GT3X than for the move II devices. The difference in bias between the two activity monitors was statistically significant (P<0.01) for all activities except for jogging (Table 3).

**Table 3:** Comparison of AEE in kcal/min for the different activities [mean (SD)].

Activity	IC [kcal/min]	Device	measure[kcal/min]	bias [kcal/min]	bias [%]	r^1^	ICC^2^	Bias: GT3X vs move II^3^
Walking	3.50 (0.58)	**move II**	3.71 (0.58)	0.21 (0.51)	6.0	0.62*	0.60*	T = 5.78; p<.01
		GT3X	4.76 (1.20)	1.26 (1.02)	36.0	0.53*	0.23*	
Fast walking	5.50 (0.96)	**move II**	5.40 (0.75)	−0.11 (0.70)	−2.0	0.69*	0.68*	T = 6.58; p<.01
		GT3X	7.23 (1.72)	1.72 (75.4)	31.3	0.70*	0.35*	
Jogging	12.41 (2.86)	move II	11.4 (2.33)	−0.93 (1.48)	−7.5	0.86*	0.80*	T = 0,94; p = .36
		GT3X	11.11 (2.38)	−1.30 (1.81)	−10.5	0.78*	0.70*	
Walking up−/downhill	5.51 (1.11)	**move II**	5.57 (0.88)	0.07 (0.53)	1.2	0.88*	0.86*	T = 5.73; p<.01
		GT3X	6.96 (1.69)	1.45 (0.96)	26.4	0.84*	0.52*	
Walking upstairs	6.57 (1.31)	**move II**	6.70 (0.78)	0.13 (0.74)	1.9	0.87*	0.77*	T = 12.54; p<.01
		GT3X	4.11 (1.12)	−2.46 (0.95)	−37.5	0.70*	0.24*	
Walking downstairs	3.56 (0.68)	**move II**	3.85 (0.74)	0.29 (0.61)	8.1	0.64*	0.60*	T = 5.72; p<.01
		GT3X	5.48 (1.69)	1.92 (1.54)	53.9	0.41*	0.14*	

*significant with p<.05.

1 pearson correlation coefficient between the device (GT3X, move II) and the reference IC.

2 intra class correlation coefficient (absolute agreement) for the device and the reference IC.

3 Paired T-Test of significance between GT3X-bias and move II-bias: Is one device more accurate? More accurate device marked bolt.

The GT3X overestimated AEE during walking, fast walking, walking up−/downhill and descending stairs and underestimated AEE for all other activities (Table 3). The largest differences between the estimated and reference value were observed while ascending and descending stairs (−2.46 (0.95) and 1.92 (1.54) kcal/min, respectively). In general GT3X underestimated moderate activity and overestimated vigorous activity.

The move II overestimated AEE while walking, walking up−/downhill and stair walking and underestimated AEE for all other activities (Table 3). The largest error in AEE was observed for jogging (−0.93 kcal/min), followed by descending stairs (0.29 kcal/min).

## Discussion

The aim of this study was to test the validity of two different activity monitors for estimating AEE for selected daily life activities against a reference method (IC). One device (GT3X) uses the commonly used single regression model for estimating AEE, and the other device (move II) first detects the type of the activity and then uses the respective regression model for estimating AEE. The latter approach was found to be more accurate for estimating AEE, which is in agreement with previous studies that proposed using different regression models according to the type [19], [24] or the intensity [25], [26] of activity to improve AEE estimation.

In general, the GT3X overestimated AEE during moderate activities (walking, fast walking, walking up−/downhill and walking upstairs) and underestimated AEE during vigorous activities (jogging and walking upstairs). This result is in agreement with previous studies that showed that single regression models overestimate EE during moderate intensity walking and underestimate EE during jogging [13] and that the GT3X significantly underestimated EE for vigorous physical activities [12]. Crouter et al. [17] tested multiple different single regressions during a wide range of activities and concluded that no single equation was appropriate for predicting AEE of all activities and that usually AEE is underestimated for most activities except for walking. Similarly, the move II overestimated AEE for most activities (that is for walking, walking up−/downhill and stair walking) and underestimated AEE for fast walking and for jogging. Similar to Berntsen et al. [12] who reported that all tested devices underestimated vigorous and very vigorous intensity physical activity, both devices in our study underestimated AEE during jogging. It can be concluded that both activity monitors were inconsistent in terms of under- or overestimating reference AEE values and that the direction of bias depends on the specific physical activity.

The largest errors in estimating AEE with the GT3X were observed for walking up- and downstairs. This is a well-known limitation of the acceleration-based activity monitors [27]: they are not able to assess the increase in energy cost of walking upstairs or uphill because the acceleration pattern remains very similar to that for normal walking despite the increased effort required to elevate the body’s center of mass, and hence tend to underestimate the AEE for these activities. In contrast, for descending stairs the acceleration magnitude is greater although the effort remains almost the same thus resulting in an overestimation of the AEE. The technology of move II not only utilizes activity-based prediction models but also comprises a barometer, and hence accounts for these differences in center of mass elevation reflected in smaller errors in estimating AEE for walking up- and downstairs than those of the GT3X device. Consequently, for activities involving elevation gain or loss, the use of barometer data for estimating AEE seems compulsory.

The move II was more accurate in estimating AEE for walking than for jogging. It is possible that the error during jogging resulted from the fact that the move II uses the same regression model for both walking and jogging. Different models for these two types of activity may generate more accurate predictions. In comparison, both the GT3X and the move II underestimated the reference AEE while crossing a sloped pedestrian bridge. One possible explanation for this underestimation is the relatively short duration of this activity and that the AEE was averaged across the entire activity. To assess the validity of the two devices for walking on an incline, walking up- and downhill should be examined separately. In addition, future studies should collect data for longer distances and hence also for longer periods of time to generate a larger and more robust data set.

This study was based on relatively small study cohort. Although we recruited subjects with different body characteristics, the final test sample included only healthy young to middle aged subjects (22 to 51 years) and therefore the results cannot be generalized for other populations (e.g. elderly people, children, obese). Moreover, the RMR-measurement for some subjects was performed in the afternoon and not in the morning as suggested by the international guidelines. However, the tests were conducted after 3 h fasting for all subjects.

In summary, the results of this study showed that the move II device was more accurate in predicting AEE than the GT3X. We conclude that using different AEE prediction models depending on the type of activity being performed improves the AEE estimation. This may be due to the fact that there is no linear relationship between the acceleration and the AEE across all different types of activities, since different types of activities include the use of different muscles. This can be modelled by using different equations for different groups of activities [28]. Furthermore the use of barometer in addition to the acceleration sensor improves the AEE estimation for the case of walking on a slope. However only a limited number of activities were examined, thus the validity of these results should be further tested for other daily activities (e.g. cycling, household activities). The result data will be made available to anyone willing to apply further statistical analysis. For this please contact the corresponding author.
